# Experiences of non-progressive and augmented labour among nulliparous women: a qualitative interview study in a Grounded Theory approach

**DOI:** 10.1186/1471-2393-7-15

**Published:** 2007-07-28

**Authors:** Hanne Kjaergaard, Anne Maria Foldgast, Anna-Karin Dykes

**Affiliations:** 1 Juliane Marie Centre for Women, Children and Reproduction, Rigshospitalet, Copenhagen University Hospital, Denmark; 2 Department of Health Sciences, Lund University, Sweden

## Abstract

**Background:**

Non-progressive labour is the most common complication in nulliparas and is primarily treated by augmentation. Augmented labour is often terminated by instrumental delivery. Little qualitative research has addressed experiences of non-progressive and augmented deliveries. The aim of this study was to gain a deeper understanding of the experience of non-progressive and augmented labour among nulliparas and their experience of the care they received.

**Methods:**

A qualitative study was conducted using individual interviews. Data was collected and analysed according to the Grounded Theory method. The participants were a purposive sample of ten women. The interviews were conducted 4–15 weeks after delivery.

**Results:**

The women had contrasting experiences during the birth process. During labour there was a conflict between the expectation of having a natural delivery and actually having a medical delivery. The women experienced a feeling of separation between mind and body. Interacting with the midwife had a major influence on feelings of losing and regaining control. Reconciliation between the contrasting feelings during labour was achieved. The core category was named Dialectical Birth Process and comprised three categories: Balancing natural and medical delivery, Interacting, Losing and regaining control.

**Conclusion:**

A dialectical process was identified in these women's experiences of non-progressive labour. The process is susceptible to interaction with the midwife; especially her support to the woman's feeling of being in control. Midwives should secure that the woman's recognition of the fact that the labour is non-progressive and augmentation is required is handled with respect for the dialectical process. Augmentation of labour should be managed as close to the course of natural labour and delivery as possible.

## Background

Non-progressive labour is the most common complication in nulliparous women. Most cases of non-progressive labour are primarily treated by augmentation with intra venous infusion of oxytocine. The incidence of augmented labour is increasing [1-4]. Augmented labour is more likely to be terminated by instrumental delivery [2,4,5]. It is estimated that 60% of all caesarean deliveries in the USA are related to non-progressive labour [3]. The triad of non-progressive labour – augmentation – instrumental delivery carries an increased risk of morbidity for the mother and baby [5-7]. In quantitative studies, non-progressive labour as well as augmented labour and instrumental delivery, are reported to have a negative influence on women's overall perception of the labour process in [8-11]. Only a few studies have explored women's experience of non-progressive and augmented labour in a qualitative design [12,13]. Further research in qualitative designs related to the birth experience of non-progressive labour is required in order to understand what initiates positive or negative experience and what will facilitate improvements of the care.

The aim of this study was to gain a deeper understanding of some nulliparas' experience of non-progressive and augmented labour and their experience of the care.

## Methods

The study was carried out as a descriptive sub study to The Danish Dystocia Study (DDS), a multi centre population based cohort study on incidence, outcome and risk factors for non-progressive labour in 2810 nulliparous women [14]. The present qualitative study was designed to gain a deeper understanding of some of the participants' experiences than is being provided through the DDS' questionnaires.

Participants were a purposive sample of nulliparous women who had recently been delivered following non-progressive labour and augmentation. The sampling aimed at achieving maximum variation and was obtained from the DDS' database. Participants' characteristics and variations according to the purposive sampling are presented in Tables 1 and 2. We exclusively invited women with a partner present at the delivery, as DDS also comprises a qualitative sub study on partners' experiences.

**Table 1:** Characteristics of participants (N = 10)

Characteristics	Number
Age groups	
- 25–31 years	7
- 32+ years	3
Educational level	
- Low education/unskilled	1
- Average education/skilled	3
- Higher education/managerial	6
Partner relations	
- Cohabiting with partner	10
- Partner present at delivery	10
Dilatation of cervix at diagnosing of non-progressive labour	
- 4 cm	2
- 5–9 cm	5
- 10 cm	3
Mode of delivery	
- Spontaneous vaginal delivery with epidural	1
- Spontaneous vaginal delivery without epidural	5
- Ventouse with epidural	3
- Caesarean delivery	1

**Table 2:** Characteristics of participants: Attitudes (N = 10)

	37 weeks of gestation	1–3 weeks post partum
Attitude towards giving birth without intervention		
- Important	8	4
- Do not know	0	3
- Not important	2	3
Attitude towards avoidance of pain during labour		
- Important	2	4
- Do not know	4	2
- Not important	4	4

The criteria for non-progressive labour were based on cervical dilatation over time in labour's first stage, active phase and descend in the second stage of labour. The criteria were based on guidelines from the Danish Society of Obstetrics and Gynaecology with regard to time limits during the first stage of labour [15,16]. We used the ACOG guideline with regard to diagnosing non-progressive labour exclusively when labour was in the active phase and to broaden the definition of arrest in second stage, descending phase, when epidural was administered (ACOG). Criteria for diagnosing non-progressive labour are given in Table 3.

**Table 3:** Definitions of stages and phases of labour and diagnostic criteria for non-progressive labour

Stage of labour	Definition of stage and phase	Diagnostic criteria for non-progressive labour
First stage	From onset of regular contractions leading to cervical dilatation to full dilatation of cervix	
Latent phase	Cervix 0 – 3 cm dilatation	The diagnosis is not given in this phase
Active phase	Cervix ≥ 4 cm dilatation	< 1/2 cm dilatation of cervix per hour, assessed over 4 hours
Second stage	From full dilatation of cervix to the child is born	
Descending phase	From full dilatation of cervix to strong and irresistible urge to push	> 2 hours without descend; if epidural is administered:> 3 hours
Pushing phase	Strong and irresistible pushing during the major part of the contraction	> 1 hour without progress

Since no invasive procedures were applied, no Ethics Committee System approval was required according to Danish law. Permission to establish the DDS database was obtained from the Danish Data Protection Agency j.nr. 2004-41-3995. Informed, oral and written consent was obtained from the participants. The policy of the Helsinki Declaration [17] was followed throughout the data collection and its analyses.

The Grounded Theory method [18] was used in collecting and analysing data. Data was collected by the use of open, individual interviews, in accordance with Kvale [19], conducted by the first author. Interviews took place in participants' homes between May and November 2005.

Participants were informed that focus of the interview was the non-progression of labour and the need for augmentation. Following an initial question, that was identical for all participants, concerning the overall birth experience, the interviews consisted of individual follow-up questions and short open-ended questions. The interviewer introduced the following subjects if the participant did not touch on them spontaneously:

Thoughts, feelings and experiences related to

- Onset of labour and admission to hospital

- The phase of labour leading up to the decision on augmentation

- Pain and pain relief during this phase

- Communication with the staff, especially the midwife and with the partner

Interviews were tape-recorded and transcribed verbatim. All interviews were coded and discussed by two researchers. When inconsistence occurred, extra codes were added to ensure diversity at this level of the analysis. Investigators' preconceptions were discussed throughout the process.

The initial open coding was followed by a four-step categorisation, using the constant comparative method in accordance with Glaser and Strauss [18]. During this process a core category emerged. To ensure that the core category was grounded in data a theoretical coding was performed. The collection of data stopped when saturation was reached. This happened during analyses of interviews number 8, 9 and 10, which accordingly were chosen to be the last interviews as no further categories emerged. A software package, NVivo QSR International, was used to assist in data management.

## Results

During the process, eleven women were invited to an interview. Ten agreed to participate. One woman refused because her husband declined. Interviews lasted around 30 minutes and took place 4–15 weeks after delivery.

### Dialectical Birth Process

The core category was named Dialectical Birth Process. It emerged for the first time after interview 3 and was reassured continuously by identical or new codes and categories during the rest of the interviews and analyses. In total, 677 codes were found and condensed into 28 sub-categories. These were reduced into 7 categories and 3 main categories. An example is given in Table 4.

**Table 4:** An example of analysis and development of categories (Inerviewee 9)

Quotation*	Codes	Sub categories	Categories	Main categories
I have been wondering if it was because I felt insecure,	Feeling insecure	Feeling of insecurity and incapacity	Losing control	Losing and regaining control
	
as soon as I saw the midwife, I had a feeling that this would not work.	Distrusting the midwife	Negative experience of midwife	Interacting with midwife	Interacting
	
I wished so much to have a natural delivery and she wanted me to lie in the couch with my legs in stirrups	Wishing a natural delivery	Basic attitude towards delivery	Wishing a natural delivery	Balancing natural and medical delivery
	
and things were so different from what I had wished and prepared for mentally.	Things different from wishes and mentally preparation	Disappointment	Losing control	Losing and regaining control
	
Well, it just made me feel insecure.	Feeling insecure	Feeling of insecurity and incapacity	Losing control	Losing and regaining control

* one continuous quotation

The concept "Dialectical Birth Process" was based on the 3 main categories: Balancing natural and medical delivery, Losing and regaining control and Interacting. The birth process included experience of a dialectical conflict between wishing for a natural delivery and accepting a medical delivery. Acceptance and satisfaction was expressed as a feeling of reconciliation after having struggled hard with physical and mental challenges.

*"I have had this basic attitude that it should all be natural [laughs]. I didn't want any intervention. I had the opinion that this was something the body would manage in its own way and in the best way. There is a reason for the body to do as it does, so I said "No thanks" [to the drip] in the beginning - - - - - - She asks me 3 or 4 times and eventually I agreed and it was a good decision, I delivered within 20 minutes [laughs]" (Interviewee 1)*.

**Figure 1:**
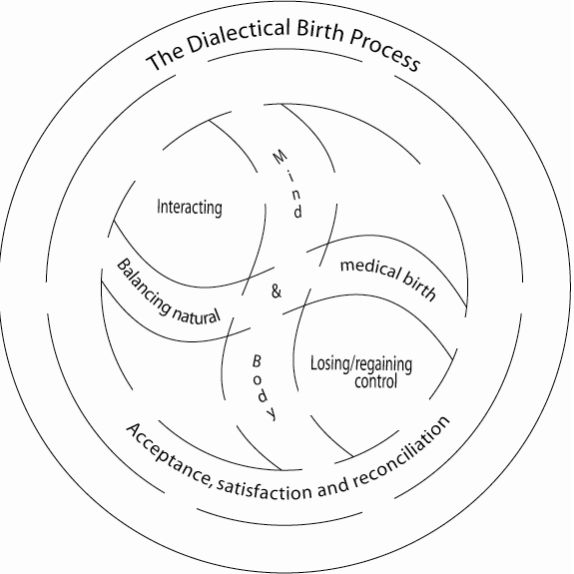
**The dialectical birth process**. The dialectical birth process comprising contrasting feelings and experiences. The two areas in the inner circle represent varying degrees of interacting and being in control. Progress of the delivery towards a natural or a medical birth and experience of separation between mind and body influence interacting and feeling of control. Acceptance followed by satisfaction and eventually reconciliation constitutes the synthesis of the process.

The concept "Dialectical Birth Process" comprises what happens in the time span from onset of labour until the establishment of augmentation and delivery. In this process several dialectically interwoven factors were identified and eventually a synthesis seemed to occur. The dialectically interwoven factors were positive and negative interaction with the midwife and the partner, experience of coherence and separation between mind and body, and losing and regaining control. Embedded in the feelings and experiences of control were in addition, positive and negative experience of pain and pain relief, participation and non- participation in decision-making regarding augmentation and pain relief. A synthesis in the process by way of reconciliation was identified. The dialectical birth process is illustrated in Figure 1. In the time span from delivery until the day of the interview reasons for non-progressive labour and future mode of delivery were considered by the participants and the perception of the birth was undergoing a change over time towards a more positive view of the overall birth experience.

### Balancing natural birth and medical birth

At onset of labour the women's desire for a delivery without medical or instrumental interaction and with as little medical pain relief as possible was pronounced. During labour this was challenged and the women faced the necessity of balancing this wish against the need for augmentation.

The process was initiated by the experience of non-progressive labour. This was expressed as a feeling of "never getting any further", a feeling of being let down by the body, being exhausted; being in severe pain and doubting that the baby would ever arrive. The feeling that the body did not help the mind and that the mind was on its own in dealing with the hard work was described vividly. The mind desired the labour to progress, but the body did not co-operate.

*"I didn't have much help from my body. I didn't have the urge [to push]. That was something I had to pull myself together to do, and so I did as good as I could" (Interviewee 4)*.

When augmentation had been established, feelings of confusion, worry and ambivalence were described along with feelings of relief and satisfaction.

*"So I had the drip and it worked well because she increased it slowly - - - I could keep pace with it and that was in fact very nice" (Interviewee 5)*.

Feelings of ambivalence were expressed, especially if an epidural was administered simultaneously. Some decisions for augmentation were closely related to the decision to have an epidural. This was a cause of concern and worry for some women.

*"I was thinking a lot about that one should not push the body, - - - I mean maybe one should listen to the body, if it doesn't want to make contractions. Especially if the body has been working well for some time and then it stops. Then it might be unnatural for the body to be pushed like that, because you give the body a heavy load of contractions at the same time as you anaesthetise it and how on earth does that affect the child, one can consider" (Interviewee 8)*.

A wish that labour and delivery would have taken another course was indicated. Although augmentation was accepted and appreciated, some women had doubts.

*"Eventually, I appreciated that drip, and I feel sorry that it has a bad reputation, that's a pity [laughs]. But I think the delivery could have taken another course - - - I should have refused to have my waters broken. I think there must be a reason why the water didn't come by itself. If I hadn't had the waters broken and I had waited 11/2 hour, he might have rotated and I might have delivered easier, a little faster and without that drip - - - It is easy to be wise after the event [laughs]" (Interviewee 1)*.

Reflections on reasons for non-progressive labour were expressed as a part of the women's views on the overall experience of labour.

*"I have been thinking that it might be because I was so tired - - -actually, I think I have been doing all the right things during pregnancy, - I have done some physical exercising, I have had wholesome food, I have stopped smoking, - - so I don't know - - - the only thing that went wrong was that I was so tired" (Interviewee 1)*.

Other reasons reflected upon by the women were overwhelming pain, the epidural or the Entonox, no help from the body, feeling insecure and unsafe or not having a good contact with the midwife. Hypothetical considerations on the possibility of another course of labour and delivery were also expressed along with reasons for non-progression.

*"My conclusion is that it [the non-progressive labour] had to do with my feeling of being insecure or unsafe - - - Had I only felt "on top" and felt that "this is totally ok, as it should be", and had I not needed to doubt if I had made a wrong decision concerning the epidural - - - - maybe things would have gone the way they should have gone" (Interviewee 2)*.

Decision-making was interwoven with acceptance and was also seen as a heavy responsibility to bear. The level of confidence in the midwife's assessment was expressed along with doubts of the necessity of the augmentation. Decision-making was mostly conducted in co-operation between the woman and the midwife. Not being involved in the decision-making was also mentioned, but did not appear to be a problem.

*"It was explained to me what the disadvantages could be. It could provoke severe pain, but I had the epidural, and just because I had that, I don't think they made a big deal of explaining to me - - - but well - It [the drip] was installed and - - I wanted the drip" (Interviewee 10)*.

At the time of the interview the overall perception of the birth experience was positive. This was expressed as a motion over time. The feeling of acceptance of the need for augmentation occurred during the course of labour. Immediately after labour a feeling of satisfaction was prevalent and eventually at the time of the interview an overall feeling of reconciliation was achieved. For some it had made sense to consider a future mode of delivery, for instance elective caesarean delivery and home delivery.

*"Well I think of it in a different way now - - - -Immediately after birth I thought that it was the worst thing I had ever experienced, I didn't see any positive conditions at all. But now I find something positive, retrospectively. There are things that I find more positive in retrospect" (Interviewee 2)*.

A couple of women described that they had no experience of non-progression or exhaustion. They mentioned that the midwife assessed the labour to be non-progressive and that they were surprised that the drip was a treatment for non-progressive labour.

### Losing and regaining control

The feeling of being in control was crucial. The women stated that they had a feeling of being in control at the onset of labour and at admission to hospital.

*"At the beginning, I felt that everything was just fine, I had been nervous for the delivery and for the pain, but in fact I handled the pain very well and I had a lot of self-confidence and felt rather strong" (Interviewee 9)*.

Losing control was related to feeling exhausted, insecure, unsafe, afraid and scared. Feeling overwhelmed by severe pain, not knowing what was going to happen or losing confidence in the midwife was also related to losing control. A perception of separation between mind and body leaving the mind incapable of controlling what happened in the body was a source of frustration.

*"I got scared because I could not control what was happening in my body. I was so tensed, my legs and arms were shivering and - - when I was speaking, my voice was trembling. One gets so frightened for something you're not able to control I think, – so that's what is happening. And furthermore, one doesn't know what is going to happen, – because anything and everything can happen. It is the unforeseeable one gets frightened for" (Interviewee 5)*.

Regaining control was related to sufficient pain relief, being able to rest, feeling well informed and being respected for subjective signs and perceptions.

*"It worked [the epidural] and that was fantastic ...I was so tired. It was an extreme relief to get rid [of the pain] and also stop vomiting. It really was wonderful ------ and I felt so optimistic. It came back when I had that epidural" (Interviewee 7)*.

Losing as well as regaining control was closely related to interaction with the midwife.

*"She had a conflict with the doctor. I clearly sensed that. She was kind of surprised or frightened, when I asked, what that was about? The conflict was evident to me. And because you try to stick to someone that you can trust and rely on, I got lost when she kind of got frustrated" (Interviewee 8)*.

### Interacting

The category is closely connected to the category "Losing and regaining control", as interaction between the woman and the midwife influences the woman's experience of control. A positive interaction included that the midwife acted as a coach and guided the woman, respecting her wishes and subjective perceptions, but also demonstrating that she, (the midwife) was the one who ultimately knew what was the best.

*"She looked at me and said: You don't look like someone, who should be sent back home, – and she was right, I really shouldn't be sent back home - - - I found that she was incredibly good at guiding me. I needed guidance and pushing a little bit all the time, but at the same time I was allowed to decide what I wanted" (Interviewee 7)*.

The midwife's professional skills, and the way she demonstrated these, were of importance for a positive interaction, which was obvious when the midwife radiated security, confidence, empathy, and commitment and when she was calm, kind and informative, also towards the husband/partner.

*"They were very kind, the three midwives that I had. They were sweet, helpful, assisting and understanding. - - - - When I should have the epidural, I remember that she was very empathic. She was looking at me, and I must have had a strange look on my face, which also she had herself indeed, and afterwards she said that she suddenly had doubts whether it was the right thing for me to have the epidural" (Interviewee 10)*.

It was stated that the most important relation and interaction was the one between the woman and the midwife, when this interaction was positive. Elements in a negative interaction with the midwife were the midwife being "invisible", uninformative, not being in control of the situation and not listening to the woman's wishes or showing her respect.

*"I felt deceived by her. I wanted to deliver standing upright next to the bed but I don't think she had ever tried that and she wasn't very happy about it. But we had agreed on it earlier - - - but then she asked me to lie on hands and knees and suddenly she asked me to turn around, - - and there I was lying on the bed although I had pointed out that I did not want to lie on my back. But then she said that I couldn't push properly if I wasn't lying in this way and that was that. It ended up like that" (Interviewee 9)*.

Experience of positive interaction included the partner's ability to offer caring and loving support, not needing support from the woman, and also being able to actively advocate for the woman's point of view. When the interaction with the midwife was negative the partner was the emotional centre point. Overall the women were satisfied with the interaction with their partner. One partner's impatience and desire for augmentation and instrumental delivery was mentioned but did not create a problem for the couple.

*"C (partner) was fantastic he was really good. He took it as a man. Yet I remember feeling that he was impatient. He kept on saying: " Shouldn't we increase the drips" [laughs] ----- He also said: "Shouldn't we use the vacuum" But then I told him off [laughs]" (Interviewee 2)*.

### Outline of a theoretical model

Two central issues emerged during data collection and analyses. The first one had its origin in expectations of a natural delivery and experience of a non-progressive labour with augmentation and, for some, also an instrumental delivery. Feelings of disappointment and frustration were expressed. The second issue appeared when a perception of the body having its own will was expressed. It was voiced that "the body can manage this", "my body would not - -" and "I didn't have any help from my body". These two issues were intermediate steps between codes and categories and were repeated and strengthened by the progressing categorisation. Eventually they contributed to the category *Balancing natural and medical delivery *and through this category they constituted the basis for an outline of a theoretical model that includes all the final three main categories and can be presented as follows: Having expectations of a natural delivery is, among other factors, based on a fundamental confidence that the body will be able to manage the physical demands of labour and delivery. During labour the body is seen as being separated from the mind and this dualism creates a conflict that makes the woman in labour feel let down by her body when her labour is non-progressive. She perceives her mind as "me" or "I" whilst her body is outside "me" or "I". Accordingly she must balance her body, which is not interacting with her mind, to her mind, which is disappointed that the expectation of a natural delivery is not being met.

In this situation the woman faces the impact of the process of *Interacting *with the midwife and the partner and the impact of the process of *Losing and regaining control*. Both of these processes hold possibilities of dialectical interplay with the potential to create reconciliation. Reconciliation is seen as a mentally healthy synthesis in the dialectical process. For the women in this study, reconciliation was the end point in an emotional motion initiated by acceptance of the need for augmentation and potential subsequent interventions, i.e. a medical delivery. A feeling of satisfaction followed acceptance immediately after the delivery and eventually reconciliation was expressed as a present feeling at the time of the interview. The midwife's handling of inter personal interaction with the woman and support of the woman's feeling of being in control has a major impact on whether a dialectical birth process will include reconciliation or not. Figure 1 illustrates the dialectical birth process that this outline of a theoretical model is based upon.

## Discussion

The core category was named Dialectical Birth Process. G W F Hegel developed the theory of dialectic in modern philosophy through the statement that harmony of thought and being can occur only through discord and that only by the working-through to a certain shape of thought, a reconciliation of thought and being can be achieved in its fullest sense [20]. More generally speaking, the term dialectical refers to a process or situation involving contradictions or conflicts of opposites and their resolution. The resolution is the synthesis in a process of thesis and anti-thesis. The term Dialectical Birth Process refers to the woman's contrasting feelings, thoughts, expectations and experiences of what is happening during labour and delivery (the "thesis" and "anti thesis") and eventually the synthesis in acceptance, satisfaction and reconciliation. Three central and interwoven categories were identified: "Balancing natural birth and medical birth ", "Losing and regaining control" and "Interacting".

### Balancing natural birth and medical birth

Feelings of disappointment, ambivalence and eventually reconciliation were elements in the woman's emotional balancing between desiring a natural delivery and experiencing a medical delivery. This echoes the findings of Carlton in a study on decision-making in labouring women [21]. Reconciliation is seen as part of mental health in other studies. Berg found, in a study on women's handling of pregnancy and diabetes, that reconciliation with having diabetes was connected with the ability for self-understanding [22]. In our study the non-progressive labour was mainly, but not solely, described as a negative experience in accordance with the findings of Nystedt in a qualitative study [8]. We found that the women's expectations had an impact on the experience. This has been voiced by others, [23-25] and underlines the importance of the midwife being cognisant of he woman's expectations. At the time of the interviews, the overall experience of the delivery was positive. It was mentioned though, that this had not been the case all the time. A change in the perception of birth experience over time has also been reported by others [26].

### Losing and regaining control

Feelings of internal and external control were expressed at the onset of labour and at admission to hospital. All but one reported losing control during labour and of these all but one regained control. In accordance with other studies, we found that several sources may endanger the feeling of being in control. In general the feeling of being in control is crucial to the woman in labour, and it is an important factor related to satisfaction with the childbirth experience and subsequent well-being [9,24,27-29].

We found that severe pain and dizziness caused by medical pain relief threatened the woman's sense of being in control. This is in accordance with others' findings [8,23,27,29,30]. A review on pain and birth experience satisfaction concludes that experience of pain and pain relief is essential when women evaluate their birth experience [24]. We had implications that feeling out of control might lead to an experience of greater pain, just as having a positive interaction with the midwife was seen to have a positive influence on the perception of pain. This underlines the interweaving of influencing factors on the sense of control that others have emphasised [27]. In accordance with Nystedt [8] we found that pain relief was essential for the feeling of regaining control.

### Separation between mind and body

To our knowledge the finding "feeling of separation between mind and body" has not previously been reported in studies on experience of non-progressive labour. The dualism mind/body had a negative influence on the woman's sense of control and strongly supported the development of our core category and the associated outline of a theoretical model. Charmaz has studied the experience of patients having fallen seriously ill, and describe that they have a perception of dualism between the sick self/physical self, which is the "I – body" and the monitoring self, which is the "I – mind", named "the dialectical self" [31]. Nystedt found that women with non-progressive labour had a feeling of having suddenly fallen ill and being in a life-threatening situation [12]. We did not find this explicitly, but the experience of separation between mind and body might have the same origin in women experiencing non-progressive labour as in people who are seriously ill, as described by Charmaz [31]. This dualism, expressed by separation between body and mind is contradictory to the concept of a lived and existential body as voiced by Merleau-Ponty who criticises the philosophical tradition, which has a tendency to consider the body simply as an object that a transcendent mind orders to perform varying functions [32]. Pregnant women's basic perception of body and mind being separated is probably influenced by the prevailing philosophy and basic view of disease in the natural sciences and Western European medicine [33,34]. Knowing that this perception of separation between body and mind might entail a negative feeling in the labouring woman of being let down by her body, the midwife should not assist this perception but stress a broader approach to the subject already during antenatal visits and prenatal classes.

### Interacting

It was essential for regaining control that the midwife was in control of the entire situation and that she was supportive, empathetic, showed respect for the woman and her partner as unique individuals and encouraged the woman to express her wishes. This is in accordance with findings in other studies [22,25,29,30,35,36]. A caring and loving interaction with the partner was important for the overall birth experience. It was important that the partner was able to take care of himself and that he did not need attention from the woman. This underlines the midwife's role in paying attention to and taking care of the partner.

Decision-making on the need for augmentation was a step towards the acceptance of a medical delivery and reconciliation. This finding echoes findings from other studies [13,21,37]. Like Blix-Lindstrøm [13], we found that some women did not participate in decision-making and that this was not crucial for them. Participation in the decision-making was mentioned among reasons for having a bad conscience towards the child because of the interventions that followed a decision and the worries about any potential harm that these interventions might cause to the baby. This raises the question of the pros and cons in labouring women's participation in decision making and has vast ethical implications that are beyond the aim of this study.

### The theoretical model

The outline of a theoretical model is based on the core category Dialectical Birth Process and the three main categories, Balancing, Losing and regaining control and Interacting. The theoretical model fits all the categories and it is considered that the study and the findings are relevant to and in accordance with real problems and needs in nulliparous women during labour. The theoretical model works in the sense that it gives an implication of how these findings interact and how they can be applied in clinical practice. This can be done by drawing midwives' attention to the dialectical process in which these women found themselves, when an expected natural course of labour and delivery turned into a non-progressive labour. The importance of the midwife's handling of interpersonal interaction and support to the woman's feeling of being in control should be explicated.

The theoretical model is modifiable and transferable, as the dialectical process is most likely to take place in the same situation even in other contexts. According to our findings and the literature, the categories Losing and regaining control and Interacting comprise issues of general relevance for other types of labour and delivery, that might include a potential conflict between expectations and the way the pregnancy, labour and delivery develops. In our study the reconciliation in the dialectical process included that the women agreed on the necessity of augmentation and also felt satisfied in this choice.

### The method

This sub study of the Danish Dystocia Study was created to constitute an explorative supplement to information on psychological issues from questionnaires in the quantitative study by elaborating on some women's experiences.

We chose the qualitative research interview and a Grounded Theory method for data collection and analysis. Our research question, as presented in the aim, could most likely have been investigated even in other qualitative designs. The Grounded Theory method was preferred; as the strength of this method is that the credibility of findings is enhanced through the constant comparison of codes and categories. This ensures that interpretation and understanding is constantly and strictly related to data. The individual interview was chosen in order to capture each woman's personal experience.

The internal validity of our study is based on the appropriateness of the design and the method, which is regarded as being well suited for obtaining information on how labour and delivery is experienced. Prior to the interview, the woman was told that we would focus the interview on the period of labour from onset of labour until non-progression was diagnosed and augmentation established. The interviews lasted around 30 minutes, which might seem a little short for a qualitative research interview. As we had access to all background information about the participants from DDS database, we did not need to spend time collecting any of this during the interviews. All three authors participated in the analyses, which might also have contributed to the internal validity.

The external validity of our findings is based on the convergence between some of our findings and other research, which suggests that our findings can be transferred to other similar populations. The fit, relevance, workability and modifiability of the theoretical model of the Dialectical Birth Process' also add to the external validity.

To our knowledge the findings of separation between mind and body in non-progressive labour and the outline of our theoretical model have not been previously described. Therefore, further research is needed for supplementation and validation of our findings.

## Conclusion

The findings in our study include characteristics of experiences of non-progressive labour and contribute to the sparse literature on this subject based on qualitative research. The central finding was that the women found themselves in a contrasting process when experiencing a non-progressive labour. To navigate in this situation, a feeling of being in control was of the greatest importance as well as experiencing a respectful and empathetic interaction with the midwife and a caring and loving interaction with the partner. By elucidating that these factors also have an impact on non-progressive labour, our study supports that midwives should pay attention to this in any course of labour and delivery. The outline of a theoretical model contributes to existing knowledge by focusing on understanding the psychological mechanisms at stake in non-progressive labour and gives the midwife guidance towards a specific handling of the care for the woman experiencing a non-progressive labour. The midwife has an important task in securing that the woman's psychological process of realising that the labour is non-progressive and that augmentation is needed, is handled with respect for the dialectical process and thus the midwife should actively assist the woman in being reconciled with a medical birth including reconciliation with her body. In addition augmentation of labour should be managed as close to the course of natural labour and delivery as possible.

## Abbreviations

DDS The Danish Dystocia Study

## Competing interests

The authors declare that no conflicts of interests are related to this study.

## Authors' contributions

HK and AKD planned this qualitative sub study of The Danish Dystocia Study. HK carried out the data collection. All three authors participated in the analyses. HK wrote the drafts of this manuscript, which AKD and AMF commented on. All three authors approved the final manuscript.

## Pre-publication history

The pre-publication history for this paper can be accessed here:


